# A newly discovered member of the Atlastin family, *BmAtlastin-n*, has an antiviral effect against BmNPV in *Bombyx mori*

**DOI:** 10.1038/srep28946

**Published:** 2016-06-29

**Authors:** Tai-hang Liu, Xiao-long Dong, Cai-xia Pan, Guo-yu Du, Yun-fei Wu, Ji-gui Yang, Peng Chen, Cheng Lu, Min-hui Pan

**Affiliations:** 1 State Key Laboratory of Silkworm Genome Biology, Southwest University, Chongqing, China; 2 Key Laboratory for Sericulture Functional Genomics and Biotechnology of Agricultural Ministry, Southwest University, Chongqing, China

## Abstract

Atlastin is a member of the dynamin protein superfamily and it can mediate homotypic fusion of endoplasmic reticulum (ER) membranes, which is required for many biological processes. In this study, a new Atlastin homologous protein, BmAtlastin-n, was characterized in silkworms and was found to contain an N-terminal conserved GTPase domain and a coiled-coil middle domain. BmAtlastin-n is localized in the cytoplasm and enriched in silkworm midgut. Results also showed that overexpression of *BmAtlastin-n* in BmN-SWU1 cells could enhance resistance to BmNPV. To better confirm its antiviral effect, microRNA was used to knock down the expression of *BmAtlastin-n* in BmE-SWU1 cells with inducing the reproduction of BmNPV. A transgenic expression vector of *BmAtlastin-n* was constructed and introduced to silkworm embryos by microinjection. The transgenic silkworm also showed considerable antiviral capacity. In conclusion, these findings demonstrate that BmAtlastin-n plays an important role in BmNPV defense. More importantly, the current study may provide a new clue for Atlastin research.

Atlastins belong to the dynamin superfamily, which is involved in several physiological functions of endomembrane in eukaryotic cells^1^, including formation of transport vesicles^2^, mitochondrial fusion and fission^3^, membrane fusion^4^, and pathogen resistance^5^. Nearly a decade ago, genetic mutation of Atlastin was identified as a cause of a form of autosomal dominant hereditary spastic paraplegia (HSP), which is characterized by progressive lower-extremity weakness and spasticity^6^. Atlastin has an N-terminal conserved GTPase domain, a three-helix-bundle (3HB) middle domain, two tandem transmembrane (TM) segments, and a C-terminal cytoplasmic tail (CT)^7^. Current studies have reported that Atlastin is required for the formation and maintenance of ER networks^8^ and mediates the homotypic ER fusion and vesicle trafficking in the ER/Golgi interface^9^. In *Drosophila melanogaster*, overexpression of Atlastin causes expansion of the ER membranes and inhibition of Atlastin results in ER fragmentation^10^. The depletion of Atlastin2/3 can cause unbranched ER tubules in Hela cells^8^; in *Xenopus* egg extracts, Atlastin antibodies inhibit formation of the ER network^11^. Atlastin-1 is highly expressed in the brain and is enriched in growth cones, also benefits axon elongation during neuronal development^12^,^13^. *Danio rerio* Atlastin also controls larval mobility and spinal motor axon growth by inhibiting the bone morphogenetic protein (BMP) pathway^14^.

Atlastin has been found widely in many different species. Some of them, such as *Homo sapiens*, *Xenopus laevis*, and *Danio rerio*, have three Atlastin isoforms. In others, such as *Drosophila melanogaster* and *Caenorhabditis elegans*, only one is present^15^. *Bombyx mori*, the silkworm, has four known Atlastin homeotic genes^16^. Silkworms are an important lepidopteran insect, and its silk is an essential biological material in both textiles and medicine^17^,^18^,^19^.

Recently, a previously undiscovered Atlastin protein was identified from the isobaric tags for relative and absolute quantitation (iTRAQ) analysis between BmN-SWU1 cells and BmN-SWU2 cells. This protein showed higher expression levels in BmN-SWU2 cells than in BmN-SWU1 cells^20^. These two cell lines, which originated from the ovarian tissue of the same silkworm strain^21^, showed different levels of susceptibility to *Bombyx mori nucleopolyhedrovirus* (BmNPV)^22^, a member of the Baculoviridae family that causes widespread epidemics of nuclear polyhedrosis (also known as “grasserie” or “jaundice”) and limits the production of silk and the development of sericulture^23^,^24^. BmN-SWU1 cells are vulnerable to BmNPV, while BmN-SWU2 cells lack the interaction between host and BmNPV during the viral entry process, which inhibits the reproduction of BmNPV^22^. Although there are no clear reports about the effects of Atlastin against various pathogens, it is here speculated that the different patterns of expression of this protein may be one of the targets for BmNPV resistance in the two cell lines.

In this study, an Atlastin homeotic gene was cloned from *Dazao* strain, identified and named *BmAtlastin-n*. To confirm speculation that BmAtlastin-n may have anti-BmNPV effects, proliferation of the virus via overexpression and interference of *BmAtlastin-n* in silkworm cells was here investigated. The results confirmed that *BmAtlastin-n* plays an important role in resistance to BmNPV infection *in vitro*. A silkworm strain overexpressing *BmAtlastin-n* was produced using transgenic technology. It showed significant resistance to BmNPV infection. Overall, these findings confirmed that *BmAtlastin-n* overexpression will inhibit the reproduction of BmNPV.

## Results

### Cloning and identification of *BmAtlastin-n*

**Figure 1:**
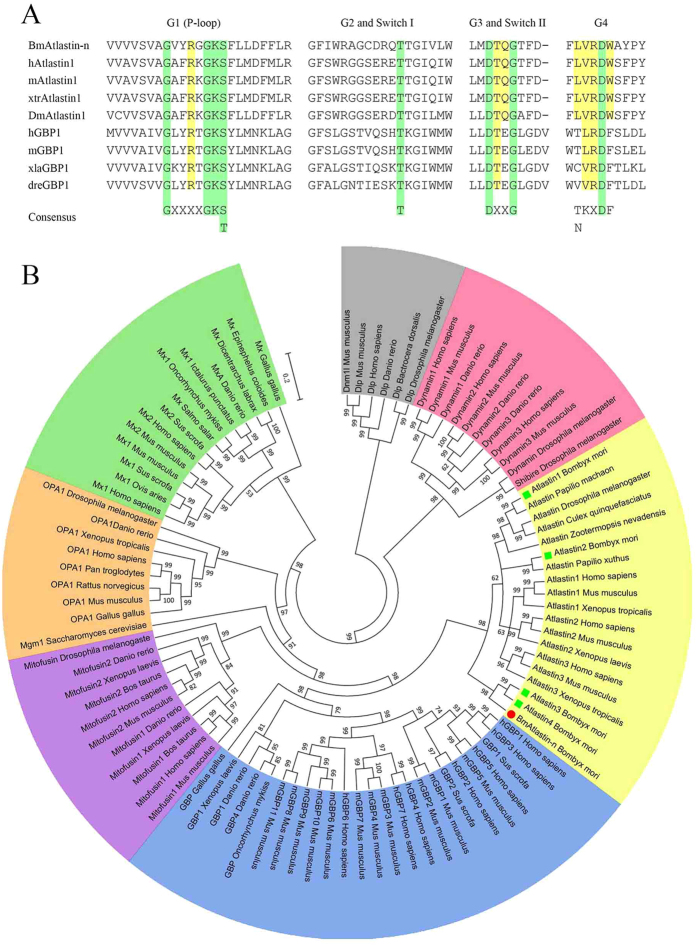
GTP binding motifs and phylogenetic analysis of BmAtlastin-n. (**A**) Conserved sequences of GTP binding motifs of Atlastins and GBPs. The Atlastin sequences shown are protein sequences of specific species, *Bombyx mori*, *Homo sapiens*, *Mus musculus*, *Xenopus tropicalis*, and *Drosophila melanogaster*. The GBP protein sequences of *Homo sapiens*, *Mus musculus*, *Xenopus laevis*, and *Danio rerio* are shown. Each sequences had four GTP-binding domains and two switch regions. The conserved key residues are shown in green and the conserved variable residues in yellow. (**B**) Bayesian phylogenetic tree of dynamin superfamily. The dynamin superfamily includes dynamins (red), dynamin-like proteins (DLP, gray), optic atrophy 1 (OPA1, orange), Mx proteins (green), Mitofusins (purple), guanylate binding proteins (GBPs, blue), and Atlastins (yellow).

*BmAtlastin-n* was cloned from larval cDNA from the *Dazao* strain, which has a 2313 bp coding sequence (CDS) encoding a 770 amino acid protein. BmAtlastin-n contained an N-terminal conserved GTPase domain and a coiled-coil middle domain but no transmembrane segments (Fig. S1). There were four conserved GTP-binding motifs, including the phosphates coordination of G1 (also known as P-loop), catalysis of G2, the formation of a hydrogen bond with the γ-phosphate of GTP of G3, and ribose coordination of G4 (Fig. 1A, Fig. S1). The two switch regions (switch I and switch II) were also observed in sequence analysis. These appeared as surface loops that would induce conformational changes upon GTP binding. Members of the dynamin superfamily were grouped using Bayesian inference method, including classical dynamins, dynamin-like proteins (DLPs), optic atrophy 1 (OPA1), Mitofusins, Mx proteins, guanylate binding proteins (GBPs), and Atlastins. Phylogenetic tree analysis showed that Atlastins and GBPs form one group and that BmAtlastin-n belongs to the Atlastin subfamily with closest relationship to silkworm Atlastin3 and silkworm Atlastin4 (Fig. 1B). These results suggested that *BmAtlastin-n* is a homologous gene of the Atlastin family.

### Location and pattern of expression of BmAtlastin-n

**Figure 2:**
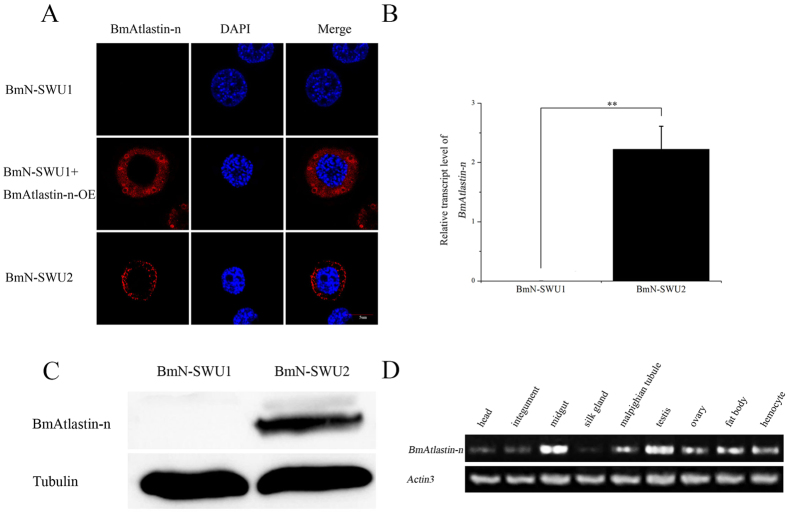
Cellular localization and expression pattern analysis of BmAtlastin-n. (**A**) The specific BmAtlastin-n antibody (anti-BmAtlastin-n) was used to detect the localization of endogenous and exogenous BmAtlastin-n in BmN-SWU1 cells and endogenous BmAtlastin-n in BmN-SWU2 cells. (**B**) The level of transcription of BmAtlastin-n was assessed in BmN-SWU1 cells and BmN-SWU2 cells using qRT-PCR (*P < 0.05, **P < 0.01). (**C**) The level of expression of *BmAtlastin-n* was analyzed using Western blotting in BmN-SWU1 cells and BmN-SWU2 cells. (**D**) The pattern of BmAtlastin-n expression was assessed in different tissues of third-day fifth-instar larvae of *Dazao* by RT-PCR (*P < 0.05, **P < 0.01) with *actin3* as a loading control.

The subcellular localization of endogenous BmAtlastin-n was detected in BmN-SWU1 cells and BmN-SWU2 cells using immunofluorescence with specific BmAtlastin-n antibody (anti-BmAtlastin-n). The laser confocal images showed BmAtlastin-n was to be located in the cytoplasm in BmN-SWU2 cells and was not detected in BmN-SWU1 cells (Fig. 2A). qRT-PCR and Western blot analysis were used to analyze the level of transcription and protein expression in order to assess the differential expression of BmAtlastin-n in the two ovarian cell lines. Results showed BmAtlastin-n exhibited high levels of expression in BmN-SWU2 cells and little or no expression in BmN-SWU1 cells (Fig. 2B,C). pIZ-BmAtlastin-n eukaryotic expression vector was constructed to determine the location of exogenous BmAtlastin-n in BmN-SWU1 cells and the exogenous BmAtlastin-n was also observed in the cytoplasm (Fig. 2A).

The pattern of *BmAtlastin-n* expression was analyzed in different tissues of third-day of fifth-instar larvae of *Dazao* by RT-PCR, including the head, integument, midgut, silk gland, malpighian tubule, testis, ovary, fat body, and hemocyte. Results showed *BmAtlastin-n* was highest transcribed in midgut and minimum in silk gland (Fig. 2D). Meanwhile, the testis, ovary, fat body, and hemocyte had higher expression levels. When BmNPV infects silkworms, the virus must cross the midgut, first cell barrier, and were further blocked by the immune tissues of hemocyte and fat body. Overall, BmAtlastin-n was located in the cytoplasm and was found to have little or no expression in BmN-SWU1 cells. BmAtlastin-n showed different transcription levels in different tissues and different patterns of expression in the two silkworm ovarian cell lines. It is here speculated that these phenotypes are caused by the BmAtlastin-n function.

### Inhibition of BmNPV reproduction via overexpression of *BmAtlastin-n*

**Figure 3:**
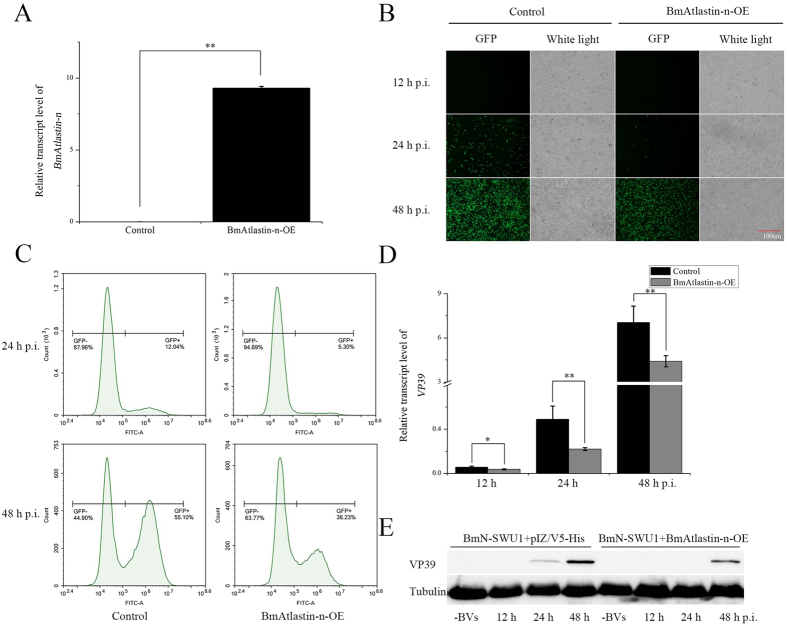
Detection of anti-BmNPV activity in *BmAtlastin-n* overexpressed BmN-SWU1 cells. (**A**) The level of transcription of *BmAtlastin-n* in BmAtlastin-n overexpressing BmN-SWU1 cells and empty vector control cells was determined using qRT-PCR (*P < 0.05, **P < 0.01). (**B**) Transfected BmAtlastin-n BmN-SWU1 cells and control cells without *BmAtlastin-n* transfected were incubated with v39K^prm^-EGFP BmNPV. Infected cells (GFP positive) were examined using fluorescence microscopy at 12 h, 24 h, and 48 h post infection. (**C**) BmNPV infection rates in BmAtlastin-n-OE BmN-SWU1 cells and control cells were analyzed by flow cytometry at 24 h and 48 h post infection. GFP-negative cells (GFP−) were uninfected and GFP-positive cells (GFP+) were infected. (**D**) Proliferation of BmNPV was assessed using qRT-PCR by analyzing relative transcription levels of *VP39* at 12 h, 24 h, and 48 h post infection (*P < 0.05, **P < 0.01). The *rpl3* gene served as an internal control. (**E**) Western blots of expression of viral late 39 K protein in *BmAtlastin-n* overexpression BmN-SWU1 cells and pIZ/V5-His-vector-transfected control cells. Tubulin protein served as the loading control.

The effects of *BmAtlastin-n* overexpression on the replication of BmNPV in BmN-SWU1 cells were analyzed, treating the *BmAtlastin-n* expression level and the differential BmNPV susceptibilities of two ovarian cell lines. First, pIZ-BmAtlastin-n was transfected into BmN-SWU1 cells and the transcriptional level of *BmAtlastin-n* was significantly higher in transfected cells (Fig. 3A). Next, v39K^prm^-EGFP reassortant budded viruses (BVs) were used to incubate BmN-SWU1 cells of *BmAtlastin-n* overexpression and pIZ/V5-His control cells, and infection states were analyzed at 12 h, 24 h, and 48 h. Fluorescence images and statistical proportions of infected cells (GFP positive) showed that *BmAtlastin-n* overexpressed cells had fewer GFP positive cells than the control cells at 24 h and 48 h after infection (Fig. 3B, Fig. S2). Because the GFP was promoted by a 39 K viral late promoter, fluorescence was not observable in infected cells at 12 h. BmNPV infection rates of the two treatments were analyzed by flow cytometry to further confirm the inhibitory effects of virus reproduction by overexpression of *BmAtlastin-n*. The virus infected (GFP positive, GFP+) cells of *BmAtlastin* overexpressing (BmAtlastin-n-OE) cells was 5.30%, which was lower than 12.04% GFP+ cells of the control at 24 h post infection. At 48 h after virus infection, the 36.23% of BmAtlastin-n-OE cells were still significantly fewer than the 55.10% of control cells (Fig. 3C). To confirm the inhibitory effects of BmNPV, the level of expression of *VP39*, which is a late baculovirus capsid gene widely used as a marker gene of BmNPV, was investigated using qRT-PCR and Western blot analysis. The data showed VP39 expression levels to be significantly inhibited in *BmAtlastin-n* overexpressing cells (Fig. 3D,E). In conclusion, overexpression of *BmAtlastin-n* in BmN-SWU1 cells was found to inhibit the reproduction of BmNPV.

### *BmAtlastin-n* interference and BmNPV proliferation

**Figure 4:**
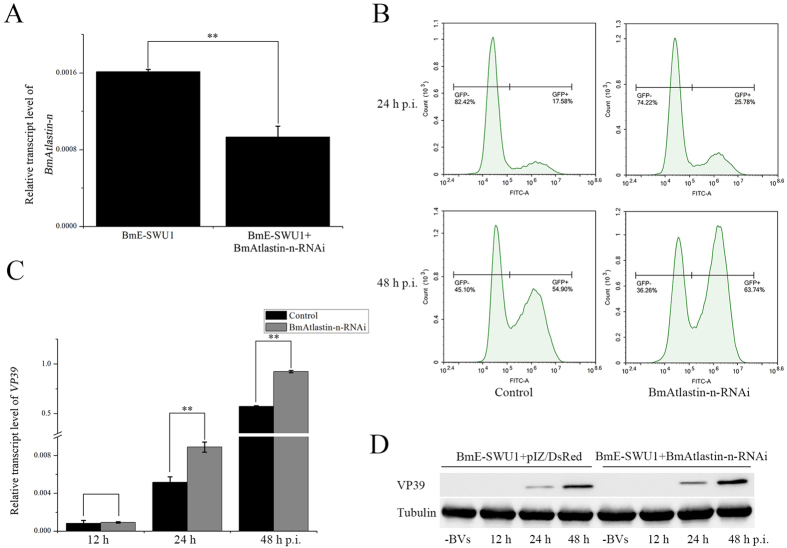
BmNPV infection and *BmAtlastin-n* interference in BmE-SWU1 cells. (**A**) qRT-PCR was used to analyze the level of transcription of *BmAtlastin-n* in BmE-SWU1 cells and *BmAtlastin-n* RNA silence BmE-SWU1 cells (*P < 0.05, **P < 0.01). (**B**) BmNPV infection rates in *BmAtlastin-n* interfered BmE-SWU1 cells and control BmE-SWU1 cells were analyzed using flow cytometry at 24 h and 48 h post infection. GFP negative cells (GFP−) were uninfected and GFP positive cells (GFP+) were infected. (**C**) Levels of *VP39* transcription were assessed at 12 h, 24 h, and 48 h after incubation of *BmAtlastin-n* interfered BmE-SWU1 cells and control BmE-SWU1 cells with v39K^prm^-EGFP. (*P < 0.05, **P < 0.01). (**D**) Induced effects on viral replication after *BmAtlastin-n* interference in BmE-SWU1 control cells and BmAtlastin-n-RNAi cells by Western blot analysis.

Because *BmAtlastin-n* overexpression enhanced the resistance of BmN-SWU1 cells to BmNPV, it is relevant whether *BmAtlastin-n* interference could induce BmNPV infection. A BmE-SWU1 cell line with high levels of *BmAtlastin-n* transcription was selected, and the expression of *BmAtlastin-n* was also inhibited by transfection with pIZ/DsRed-BmAtlastin-n-RNAi plasmids (Fig. 4A). v39K^prm^-EGFP reassortant BVs were used to incubated with *BmAtlastin-n* interfered BmE-SWU1 cells and control BmE-SWU1 cells. The data collected from flow cytometry analysis showed that the number of GFP+ cells increased in response to *BmAtlastin-n* interference in BmE-SWU1 cells. The proportions of BmAtlastin-n-RNAi cells at 24 h and 48 h post infection were 25.78% and 63.74%, which were more than the control values, 17.58% and 54.90% (Fig. 4B). Next, the transcription level of *VP39* was determined for confirmation of the proliferation of the virus (Fig. 4C). The data showed *VP39* transcript levels was significantly increased by interference of *BmAtlastin-n* at 12 h, 24 h, and 48 h after virus infection. The VP39 protein expression of the two groups was investigated further, and the results also indicated that level of VP39 expression have increased (Fig. 4D). These results suggested that *BmAtlastin-n* interference can induce reproduction of BmNPV in BmE-SWU1 cells.

### Generation of *BmAtlastin-n* overexpression in transgenic silkworms

**Figure 5:**
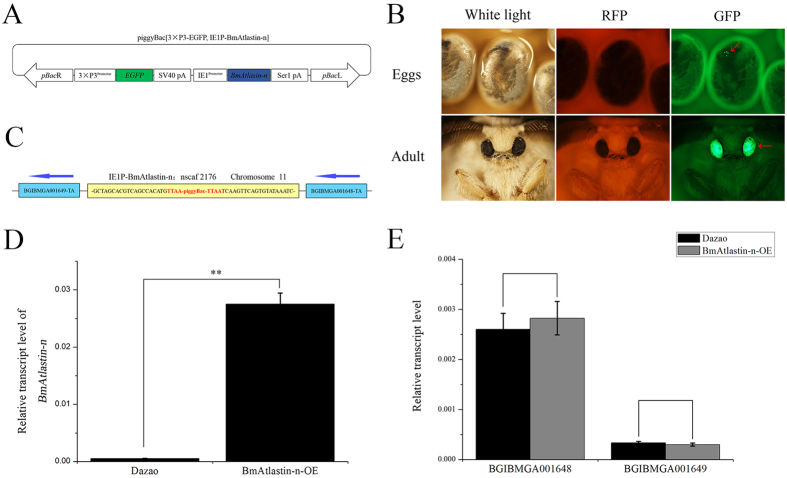
Generation of BmAtlastin-n-OE transgenic silkworm. (**A**) Schematic diagram of piggyBac [3 × P3-EGFP, IE1P-BmAtlastin-n] transgenic vector. BmAtlastin-n was driven using IE1 promotor and ser1pA was used for termination signals of transcription. (**B**) Images of positive transgenic silkworm of embryos and adults in white, red, and green lights. GFP-positive signals are shown in red arrows in the eyes. (**C**) Insertion sites in BmAtlastin-n-OE transgenic silkworms were analyzed using inverse PCR. The adjacent genes of insertion sites were analyzed in silkDB (http://www.silkdb.org/silkdb/). (**D**) Analysis of transcription levels of *BmAtlastin-n* in BmAtlastin-n-OE transgenic silkworm and *Dazao* control silkworm by qRT-PCR (*P < 0.05, **P < 0.01). (**E**) Transcription levels of *BGIBMGA001648* and *BGIBMGA001649* in BmAtlastin-n-OE transgenic silkworm and *Dazao* control silkworm (*P < 0.05, **P < 0.01).

It was here determined that *BmAtlastin-n* overexpression can suppress the reproduction of BmNPV *in vitro*. *BmAtlastin-n* was overexpressed in individuals to confirm its antiviral effects. PiggyBac [3 × P3-EGFP, IE1P-BmAtlastin-n] transgenic vector, *BmAtlastin-n* controlled by IE1 promotor, and Ser1 polyA termination signals of transcription were constructed (Fig. 5A). The transgenic vector piggyBac [3 × P3-EGFP, IE1P-BmAtlastin-n] and the helper vector pHA3PIG were co-injected into *Dazao* embryos to generate G0 offspring. G0 moths were inbred or backcrossed to product G1 progeny. Transgenic silkworms were produced via screening for GFP-positive expression from embryos and months of same brood of G1 (Fig. 5B). Inverse PCR was used to analyze the insertion site, and the PCR products were cloned and sequenced. Results indicated that fragments were inserted in the genome successfully, and there was only one insertion site. It was located on nscaf 2176 of chromosome 11^th^ (Fig. 5C).

The level of expression of *BmAtlastin-n* was detected in BmAtlastin-n-OE transgenic silkworm and *Dazao* control silkworm. qRT-PCR data suggested that *BmAtlastin-n* had been successfully overexpressed in transgenic lines (Fig. 5D). To confirm that the expression of nearest genes *BGIBMGA001648* and *BGIBMGA001649* to the left and right of *BmAtlastin-n* insertion site were affected in BmAtlastin-n-OE transgenic line, the level of transcription of the two genes was analyzed in two silkworm lines. Results showed there to be no significant difference (Fig. 5E). *BGIBMGA001648* was predicted to encode ferrochelatase, a mitochondrial-like protein. No proteins showed similarity to *BGIBMGA001649* under blast analysis. These results indicated that a BmAtlastin-n-OE transgenic line had been produced. This line could be used for next experiments.

### Enhancement of anti-BmNPV capacity in BmAtlastin-n-OE transgenic line

**Figure 6:**
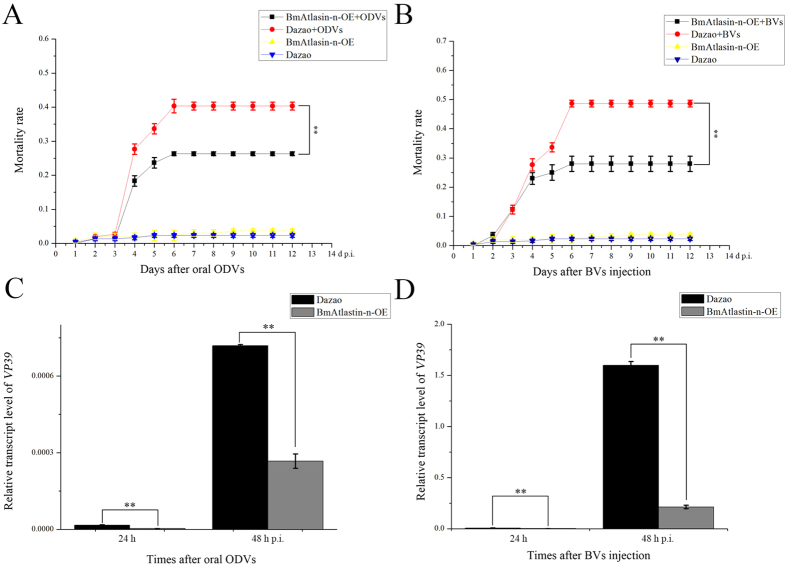
Anti-BmNPV activity of BmAtlastin-n-OE transgenic silkworms. (**A**) Mortality rates of silkworm larvae after ODVs oral infection. Each experiment set four treatments, ODV-infected BmAtlastin-n-OE transgenic silkworms, ODV-infected *Dazao* control silkworms, uninfected BmAtlastin-n-OE transgenic silkworms, and *Dazao* control silkworms. Each treatment included 100 silkworms. Mortality rates of the four treatments were determined 12 days after oral ODV (*P < 0.05, **P < 0.01). (**B**) Mortality rates of silkworm larvae after BV infection. Each experiment had four treatments, BV-infected BmAtlastin-n-OE transgenic silkworms, BV-infected *Dazao* control silkworms, uninfected BmAtlastin-n-OE transgenic silkworms, and *Dazao* control silkworms. Each treatment included 100 silkworms. Mortality rates of the same four treatments was counted in 12 days after BVs injection (*P < 0.05, **P < 0.01). (**C**) Analysis of *VP39* transcription levels in BmAtlastin-n-OE transgenic silkworms and *Dazao* control silkworms at 24 h and 48 h after oral ODVs (*P < 0.05, **P < 0.01). (**D**) Analysis of *VP39* transcription levels in BmAtlastin-n-OE transgenic silkworms and *Dazao* control silkworms at 24 h and 48 h after BV injection (*P < 0.05, **P < 0.01).

To investigate the anti-BmNPV capacity of the BmAtlastin-n-OE transgenic line, first-day fourth-instar larvae of the BmAtlastin-n-OE transgenic silkworm and *Dazao* control silkworm were infected with BmNPV via occlusion-derived viruses (ODVs) oral infection and BV infection. Each larva was fed 1 × 10^6^ ODVs and injected with 1 × 10^6^ BVs per larva. Then, mortality statistics were calculated each day until the 12^th^ day after infection. The oral ODV mortality of *Dazao* silkworm and BmAtlastin-n-OE transgenic silkworm was 40.3% and 26.3%, respectively, and the BV infection groups of *Dazao* silkworm and BmAtlastin-n-OE transgenic silkworm were 48.7% and 28% (Fig. 6A,B). The BmAtlastin-n-OE transgenic line had lower mortality in both oral infection and BVs injection groups than the *Dazao* line.

To further confirm the anti-BmNPV capacity of BmAtlastin-n-OE transgenic line, *VP39* expression levels were analyzed 24 h and 48 h after oral ODV and BV injection. The qRT-PCR results suggested that overexpression of BmAtlastin-n inhibited the reproduction of BmNPV *in vivo* (Fig. 6C,D). The BmAtlastin-n-OE transgenic line was found to have significantly less reproduction of BmNPV.

## Discussion

BmAtlastin-n was identified as a highly expressed protein in BmN-SWU2 cells using iTRAQ analysis of two cell lines. BmAtlastin-n formed a group with Atlastins and contained a GTPase domain, a coiled-coil middle domain and a C-terminal domain with no known function (Fig. S1). This is the fifth Atlastin homologous gene to be found in silkworms. However, it lacked the typical transmembrane segments of Atlastins, silkworm Atlastin3 and Atlastin4 also lacked. These three Atlastins could form the same branch. The other two silkworm Atlastins, Atlastin1 and Atlastin2, possessed characteristics typical of the Atlastin family, containing the typical transmembrane segments. They showed high homology with *Drosophila melanogaster* Atlastin and other entomic Atlastins. Atlastins and GBPs had a very high homology, sharing an N-terminal conserved GTPase domain, and they may have arisen from a common ancestor^5^. In the present study, no GBPs were found in the genomes of silkworms, *Ciona*, sea urchin, Drosophila, or *C. elegans*^25^. For these reasons, BmAtlastin-n was placed in the Atlastin subfamily.

The pattern of expression of *BmAtlastin-n* was investigated in two ovarian cell lines that the results were consistent with iTRAQ analysis. The levels of expression in tissues showed BmAtlastin-n to be enriched in the midgut. BmAtlastin-n was located in the cytoplasm in two ovarian cell lines (Fig. 2A). Whether it is also located in intracellular membranes like the Atlastins of other species needs further research. Atlastins are a subfamily of the dynamin superfamily, and recent work has focused mainly on why Atlastin mutations cause HSP and other effects in the endomembrane. However, there were no clear reports that Atlastins could take part in host innate immunity. It was here confirmed that *BmAtlastin-n* overexpression could inhibit the reproduction of BmNPV both *in vitro* and *vivo*, and the interference of *BmAtlastin-n* was found to promote viral replication in BmE-SWU1 cells (Figs 3 and 6). However, there were no effects on viral infections via interfering *BmAtlastin-n* in BmN-SWU2 cells (Fig. S3). It was here speculated that BmN-SWU2 cells lack the entry mechanisms of BmNPV, causing the interference of *BmAtlastin-n* to have no effect on virus infection. In a previous study, results showed the entrance of BmNPV to be triggered by overexpressing *BmREEPa* in BmN-SWU2 cells^20^. However, the BmNPV that entered the BmN-SWU2 cells was still inhibited. This phenomenon suggested that there were other factors involved in resisting the proliferation of BmNPV inside the cells and *BmAtlastin-n* may be one of those factors. To explain why *BmAtlastin-n* exhibited an anti-BmNPV function, the fact that Atlastins and GBPs showed high homology and that GBPs plays an important role in resistance to many pathogens were considered. The functions of GBPs in the immune defense against *Chlamydia trachomatis*^26^, vesicular stomatitis virus, encephalomyocarditis virus^27^, influenza A virus^28^, Coxsackie virus^29^, hepatitis C virus^30^, dengue virus^31^, and *Toxoplasma gondii*^32^ have been characterized. Results also showed Atlastins to be similar to GBPs from the results of phylogenetic tree analysis (Fig. 1B). Taken together, these results suggested that BmAtlastin-n has functions similar to GBPs in the evolutionary process. However, the anti-BmNPV mechanism of *BmAtlastin-n* remains unknown.

Overexpression of an endogenous or exogenous antiviral gene is an effective method of improving the antiviral capacity of silkworms using transgenic technology^33^. However, most of the studies of antiviral genes are performed *in vitro* level, and any antiviral effects must be validated in the silkworms. In the current study, a BmAtlastin-n-OE transgenic line was generated via embryo microinjection. The transgenic silkworms reduced 21% mortality than controls after BVs injection and 14% mortality after oral infection (Fig. 6A,B). These results demonstrated that overexpression of *BmAtlastin-n* could enhance silkworm resistance to BmNPV. Previous studies have shown that the transgenic silkworm LI-A of *Bmlipipase* overexpression could improve the 33% survival rate after oral infection with BmNPV and the transgenic silkworm HEKG-B by overexpression of *hycu-ep32*, which was controlled by hr3 combined with 39 KP. The mortality of HEKG-B was then ≈30% lower than in controls^34^,^35^. The antiviral capacity of BmAtlastin-n-OE transgenic line was slightly lower than in these transgenic strains. This may have been caused by the differences in the promoter or strain. More importantly, the current study provides material for anti-BmNPV cultivation of silkworms that may facilitate functional research of silkworm Atlastins.

This is the first study to show that the homeotic gene of Atlastins plays a role in innate immunity of silkworms and may provide new insights into the determination of Atlastin function. Silkworm Atlastin3 and Atlastin4 showed more homology with BmAtlastin-n, which lacked the typical transmembrane segments. More studies will be required to confirm whether they also have anti-BmNPV capacity.

## Methods and Materials

### Silkworm strain, cell cultures and virus

The silkworm strain *Dazao* was maintained at the Gene Resource Library of Domesticated Silkworm of Southwest University, Chongqing, China. The tissues were isolated from third-day fifth-instar *Dazao* larvae. Two ovarian cell lines, BmN-SWU1 and BmN-SWU2, were cultured in 27 °C with TC-100 insect medium. The BmE-SWU1 embryo cell line was cultured at 27 °C with Grace’s insect culture medium. The recombinant BmNPV v39K^prm^-EGFP was constructed by Bac-to-Bac Baculovirus Expression System (Invitrogen, Carlsbad, CA, US.) as described previously^36^.

### Vector construction

*BmAtlastin-n* was amplified with primers (forward 5′ CCATATTCAACGGCGAAGTC 3′ and reverse 5′ ACATTAAGGATAGGCGAGCA 3′) and cloned into pMD19-T vector (Takara, Dalian, China). The correct fragments were obtained by PCR using the primers (forward 5′ cgggatccATGAGCAGTCTCGGTGAAGC 3′ and reverse 5′ ccctcgagCGTATACCGAACTCACGAGTG 3′) from pMD19-BmAtlastin-n. The second products and insect expression vector pIZ/V5-His (Invitrogen) were ligated using BamH I and Xho I sticky ends and constructed final vector pIZ-BmAtlastin-n-OE.

Vector pIZ/DsRed-BmAtlastin-n-RNAi contains a DsRed fluorescent protein gene fused with pIZ/V5-His vector and silkworm endogenous microRNA backbones: bom-mir-279^36^ for BmAtlastin-n-RNAi target sequences construction and expression. The artificial synthetic siRNA duplex sequences targeting the *BmAtlastin-n* gene replaced the endogenous siRNA targeted sequences of bmo-mir-279 and the siRNA sequences of *BmAtlastin-n* were selected using Invitrogen BLOCK-iT™ RNAi Designer (http://rnaidesigner.thermofisher.com/rnaiexpress/setOption.do?designOption=mirna&pid=7795643179598091088). Then the whole sequence was synthesized by GenScript Corporation (Nanjing, China). Finally the BmAtlastin-n-RNAi sequence was sub-cloned into pIZ/DsRed.

IE1 promoter and Ser1 polyA termination signal were preserved in the laboratory. Then the two elements were added to the vector pSL1180. The *BmAtlastin-n* fragments were collected from pMD19-BmAtlastin-n by PCR with primers (forward 5′ ccctcgagATGAGCAGTCTCGGTGAAGC 3′ and reverse 5′ cgggatcc TCATATACCGAACTCACGAGTG 3′), and the products and pSL1180-IE1P-Ser1pA vectors were digested with Xho I and BamH I. The vector pSL1180-IE1P- BmAtlastin-n-Ser1pA was obtained and sequenced for constructing the transgenic vector piggyBac [3 × P3-EGFP, IE1P-BmAtlastin-n]. The fragments IE1P-BmAtlastin-n-Ser1pA were digested from vector pSL1180-IE1P-BmAtlastin-n-Ser1pA and adding to the piggyBac [3 × P3 EGFP afm].

### Cell transient transfection

Before transfection, the highly pure plasmids were prepared using Plasmid Mini Kits (Qiagen, Germany). Cells in log phase were cultured in 6-well plates or 24-well plates for approximately 24 h before transfection. Each well was transfected with plasmids and X-treme GENE HP DNA Transfection Reagent (Roche) mixture, which was mixed in 200 μl antibiotic-free and serum-free medium according to manufacturer’s instruction. Transfection medium was removed and medium containing antibiotic and serum were added after 6 h. The mixture was then cultured for 72 h.

### Quantitative real time-PCR (qRT-PCR)

Total RNA was purified from each samples using Total RNA Kit II (OMEGA) and reverse transcribed into cDNA. The specific primers of *BmAtlastin-n* (forward 5′ TCTATGGTCGGAACCGATTGT 3′ and reverse 5′ GAACTTGGAACGCTGCCTCA 3′), *VP39* (forward 5′ CTAATGCCCGTGGGTATGG 3′ and reverse 5′ TTGATGAGGTGGCTGTTGC 3′) were used for Q-PCR. The primers of house-keeping gene, *ribosomal protein gene* (*rpl3*) (forward 5′ CGGTGTTGTTGGATACATTGAG 3′ and reverse 5′ GCTCATCCTGCCATTTCTTACT 3′), *Action3* (forward 5′ AACACCCCGTCCTGCTCACTG 3′ and reverse 5′ GGGCGAGACGTGTGATTTCCT 3′), were used as internal gene. Q-PCR was carried out in a 15 μl reaction mixture (1 μl of cDNA, 0.5 mM of each primer and 2× iTaq^TM^ Universal SYBR Green Supermix (Bio-Rad) in each well of 96-well plate. The reaction conditions were 94 °C for 30 s, followed by 40 cycles at 95 °C for 5 s and 60 °C for 15 s. Then the melt curve was analyzed from 65 °C to 95 °C at 0.5 °C increments of 5 s each. The data were analyzed as described in the statistical analysis section.

### Preparation of BmAtlastin-n antibody (anti-BmAtlastin-n)

The primers (forward 5′ cgggatccAGTCTCGGTGTGAAGCCAAAGG 3′ and reverse 5′ cggaattcCGCCTTGTTGTGGAGTGAATC 3′) were used to amplify fragments from vector pMD19-BmAtlastin-n, and then constructed with pET32a-c (+) (Novagen). Vector pET32a-BmAtlastin-n was transformed into BL21 and induced by 0.5 mM IPTG at 37 °C for 5 h. The recombinant protein was purified by Ni affinity chromatography and the products were used for the immunization of New Zealand white rabbits (0.4 mg/each). Immune treatments were performed once per week for a total of four times, and then the serum was collected to prepare antibody of BmAtlastin-n.

### Western blotting

Cells were lysed with lysis buffer (Beyotime, China) followed by washing twice with PBS and denatured at 100 °C with 5 × SDS-PAGE Loading Buffer (Beyotime). Proteins were subjected to 12% SDS–PAGE and the bands were transferred to PVDF membranes. They were then incubated with primary antibody anti-BmAtlastin-n/anti-VP39/anti-α-Tubulin (Beyotime) (1/5000, 1 h, 25 °C) and secondary antibody HRP conjugated anti-rabbit IgG/HRP conjugated anti-mouse IgG (Beyotime). The final results were analyzed with ECL Western Blotting Detection System (Bio-Rad).

### Immunofluorescence

Cells were fixed with 4% paraformaldehyde for 15 min at 25 °C and permeated in 0.5% Triton X-100 (10 min, 25 °C), then blocked with Immunol Staining Blocking Buffer (Beyotime, 1 h, 37 °C). Cells were incubated with anti-BmAtlastin-n primary antibody (1:200, 1 h, 37 °C). Alexa Fluor 555 conjugated donkey anti-rabbit polyclonal (Beyotime) was used as the secondary antibody. Then the cells were imaged under a laser scanning confocal microscope (Olympus).

### Microinjection and screening

The transgenic vector piggyBac [3 × P3-EGFP, IE1P-BmAtlastin-n] and the helper vector pHA3PIG were co-injected into the silkworm eggs within 4 h of spawning using a microinjector (Eppendorf)^37^,^38^. Then the G0 silkworms were reared at 25 °C. The G1 silkworms were produced by inbreeding and backcrosses of G0. BmAtlastin-n-OE transgenic silkworms were selected in G1 broods using a fluorescent stereomicroscope (Olympus), which were found to be EGFP positive in their compound eyes.

### Analysis of the insertion site

The genomic DNA was extracted from BmAtlastin-n-OE transgenic silkworm and digested with Hae III for 16 h at 37 °C, then self-ligated in solution I (Takara). The products were used for performing the inverse PCR with the primers pBacL (forward 5′ ATCAGTGACACTTACCGCATTGACA 3′ and reverse 5′ TGACGAGCTTGTTGGTGAGGATTCT 3′) and pBacR (forward 5′ TACGCATGATTATCTTTAACGTA 3′ and reverse 5′ GTACTGTCATCTGATGTACCAGG 3′)^34^ and the fragments were cloned into pMD19-T vector (Takara) and sequenced. The results were analyzed in silkDB (http://www.silkdb.org/silkdb/).

### Oral inoculation and injection of BmNPV

Fresh mulberry leaves were cut into circles 1 cm in diameter, then each piece was smeared with 1 × 10^6^ ODVs. First-day fourth-instar larvae of BmAtlastin-n-OE transgenic silkworm and *Dazao* control silkworm were fed mulberry leaves with ODVs (one piece/larva). The silkworms, which each ate a whole piece of mulberry leaf, were collected for assessment of the mortality rate. Then a capillary tube was used to inject 1 × 10^6^ BV particles/larva in the two lines form valve. Each treatment was performed in triplicate, and each replicate included 100 larvae.

### Statistical analysis

The student’s *t*-test was used to assess any statistically significant differences between treatments. *P*-value < 0.05 was considered significant, here indicated with “*” and *P*-value < 0.01 was considered very significant, here indicated with “**”. Data from three independent experiments are here presented as means ± SEM.

## Additional Information

**How to cite this article**: Liu, T.- *et al*. A newly discovered member of the Atlastin family, *BmAtlastin-n*, has an antiviral effect against BmNPV in *Bombyx mori*. *Sci. Rep.* **6**, 28946; doi: 10.1038/srep28946 (2016).

## Supplementary Material

Supplementary Information
