# Supplementary material for: A newly discovered member of the Atlastin family, BmAtlastin-n, has an antiviral effect against BmNPV in Bombyx mori
Source: Sci Rep. 2016 Jun 29;6:28946. doi: 10.1038/srep28946 (PMC4926086; doi:10.1038/srep28946)
Supplement: Supplementary Information [file srep28946-s1.pdf]

A newly discovered member of the Atlastin family, *BmAtlastin-n*, has an antiviral effect against BmNPV in *Bombyx mori*

Tai-hang Liu[1], Xiao-long Dong[1], Cai-xia Pan[1], Guo-yu Du[1], Yun-fei Wu[1], Ji-gui Yang[1], Peng Chen[1], Cheng Lu[1, 2*], Min-hui Pan[1, 2*]

[1]State Key Laboratory of Silkworm Genome Biology, Southwest University, Chongqing, China

[2]Key Laboratory for Sericulture Functional Genomics and Biotechnology of Agricultural Ministry, Southwest University, Chongqing, China

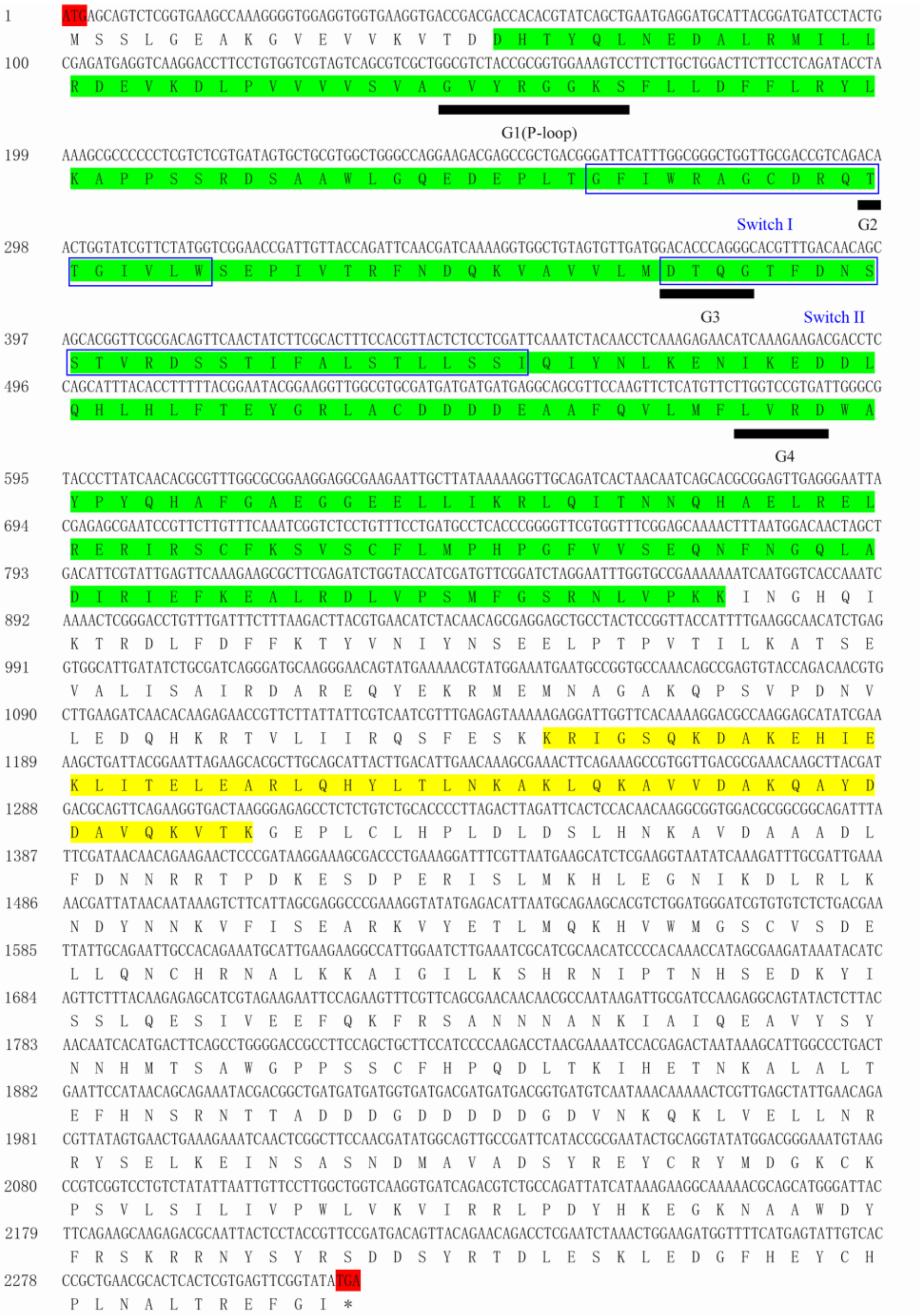

Supplementary Figure 1. Sequences analysis of BmAtlastin-n. Nucleic acid and amino acid sequences of BmAtlastin-n. BmAtlastin-n containing amino-terminal GTPase domain (yellow), middle coiled coil region (green), and carboxyl-terminus. The amino-terminal GTPase domain (yellow) contains four GTP-binding domains (G1–G4, black) and two switch regions (Switch I-Switch II, blue).

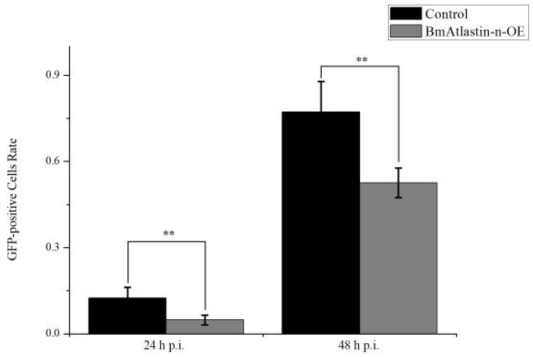

Supplementary Figure 2. Statistical rate of infected cells (GFP positive). BmNPV infection rates in BmAtlastin-n-OE BmN-SWU1 cells and control (* *P*<0.05, ** *P*<0.01).

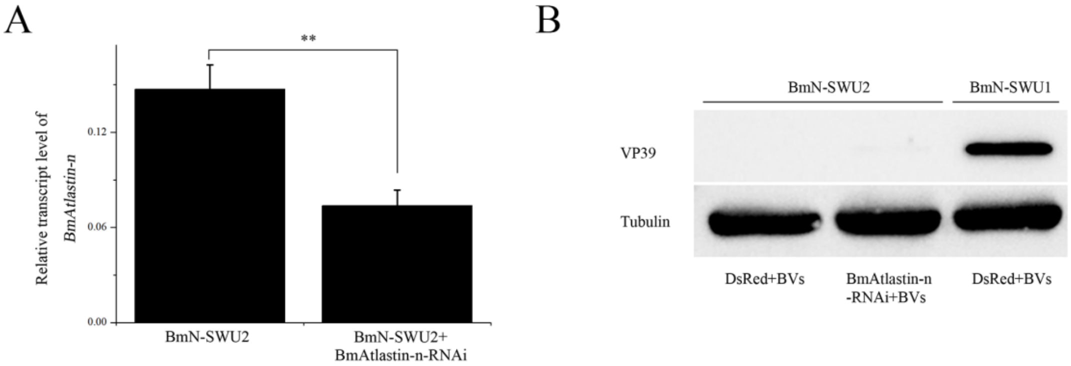

Supplementary Figure 3. BmNPV infection inand BmAtlastin-n interfered BmN-SWU2 cells. (A) Investigation of transcript level of BmAtlastin-n in BmAtlastin-n RNA interfered with BmN-SWU2 cells, as indicated by qRT-PCR (* P<0.05, ** P<0.01). (B) The proliferation of BmNPV was investigated by western blot in BmN-SWU2 cells, *BmAtlastin-n* RNA interfered BmN-SWU1 cells and BmN-SWU1 cells at 48 h post infection.
